# An online stigma reduction education program for healthcare workers working with people who inject drugs: Development

**DOI:** 10.1371/journal.pone.0347309

**Published:** 2026-04-24

**Authors:** Sandy Ezepue, Afolasade Fakolade, Tim O’Shea, Setareh Ghahari

**Affiliations:** 1 Hamilton Urban Core Community Health Centre, Hamilton, Ontario, Canada; 2 Queen’s University, Kingston, Ontario, Canada; 3 Holistique Public Health, Hamilton, Ontario, Canada; 4 Shelter Health Network, Hamilton, Ontario, Canada; 5 Hamilton Health Sciences, Hamilton, Ontario, Canada; 6 McMaster University, Hamilton, Ontario, Canada; University of New Mexico Health Sciences Center, UNITED STATES OF AMERICA

## Abstract

Canada has a high prevalence of people who inject drugs (PWID), many of whom experience limited access to necessary diagnosis, treatment, and support. PWID often avoid seeking care due to stigma from healthcare workers (HCWs). Implementing a stigma reduction education program for HCWs could improve healthcare outcomes for PWID by fostering compassionate, evidence-based approaches to care, enabling HCWs to overcome biases and deliver non-judgmental care. The objective of this study is to determine the content and format for the development of an online stigma reduction education program for HCWs working with PWID. To accomplish that, we completed interviews with PWID and focus groups with HCWs to obtain input about the content and format for the development of an online stigma reduction education program. PWID provided input on the content of the education program, highlighting the importance of module topics through personal accounts of encountering judgment and discrimination in healthcare settings, which led to their reluctance to seek available services due to fear of stigma. HCWs who participated in the focus groups provided input on the content and reached a consensus regarding the format to inform the program development. Through feedback, participants contributed to shaping the content and format, informing the development of a stigma reduction education program. These findings supported the development and refinement of a program prototype to guide future feasibility and effectiveness evaluations.

## Introduction

The global prevalence of drug use has increased in recent years. Canada has one of the highest rates of injection drug use (IDU), contributing 3.5 times the world average [1]. An estimated 171,900 individuals aged between 16 and 64 years old engaged in IDU in Canada in 2016, representing a prevalence rate of 0.7% [2]. Among the myriad risks experienced by people who inject drugs (PWID), the risk of contracting diseases such as Tuberculosis, Human Immunodeficiency Virus (HIV), hepatitis B and C, and skin and soft tissue infections (SSTI) is significantly high.

SSTI represent a significant health concern for PWID, often resulting from injecting foreign pathogens into the body through contaminated needles. Common SSTI among PWID include cellulitis, abscesses, and skin ulcers, which can lead to severe complications if left untreated, potentially resulting in fatalities [3]. However, accessing appropriate medical care for SSTI can be impeded by the pervasive stigma encountered by PWID within healthcare settings.

Emerging evidence indicates that stigma in the context of SSTI care among PWID manifests in distinct way compared to general substance use. PWID frequently delayed or avoided seeking treatment for SSTI, self drainage procedures by nonmedical individuals, use of non prescribed antibiotics, and leaving hospitals against medical advice due to fears of inadequate pain management and unmanaged withdrawal symptoms [4]. If left untreated, complicated SSTIs can lead to severe complications such as tissue damage, tissue necrosis, systemic sepsis and abscess formation, necessitating hospitalization [5,6]. Similarly, studies involving PWID have documented experiences of being treated with disdain and feeling dehumanized in healthcare settings, therefore, further promoting internalized stigma [7,8]. The presence of a trusted healthcare provider toward drug related issues has been associated with lower SSTI prevalence [4]. Initiatives that specifically enhance provider empathy and understanding in the context of SSTI management have been identified as a potentially effective strategy to improve care engagement and outcomes for PWID [6]. In response to this gap, the present study developed an online stigma reduction education program tailored to HCWs providing care for PWID with SSTI.

Stigmatizing attitudes of HCWs toward PWID can stem from various factors, including a lack of awareness about health-related stigma, misconceptions about PWID and harm reduction, and fear of infection [9]. These attitudes exacerbate the stress experienced by PWID and deter them from seeking necessary medical care, leading to delayed treatment and adverse health outcomes [10]. Education has been advocated as a powerful tool for challenging stigmatizing group stereotypes and fostering social change by dispelling myths and promoting fact-based awareness [11,12].

Development and delivery of educational interventions for HCWs are crucial to mitigate stigmatizing and discriminatory attitudes toward PWID. The delivery format of such interventions, whether in-person, online, or blended learning, can influence effectiveness. Online education programs offer a flexible and accessible approach that can cater to the schedules of many HCWs, while incorporating interactive elements to enhance learning outcomes [13–15].

The effectiveness of online programs in reducing stigma among HCWs is well-documented in the literature [9,16]. Leveraging technology for HCWs education positively impacts knowledge transfer and learning outcomes [17,18]. However, despite the availability of various online educational tools, no such program exists that is specifically tailored to reduce stigma against PWID who access SSTI services. For example, in Canada, organizations such as the Canadian Centre on Substance Use and Addiction (CCSA), the Community Addictions Peer Support Association (CAPSA), and the Mental Health Commission (MHCC) are major contributors to the development of free, readily accessible, and interactive online stigma reduction education programs. However, a review of these Canadian education programs reveals a gap in their content; the program content is frequently broad in scope, focusing on mental illness and substance use disorder, which limits its applicability to specific health conditions such as Injection Drug Use (IDU) and SSTI (CCSA, 2024; CAPSA, 2024; MHCC, 2024). To address this gap, this study aimed to develop an online stigma reduction education program to equip HCWs working with PWID with the knowledge and skills necessary to reduce the stigma associated with IDU. By filling this gap in existing resources, this program has the potential to improve access to SSTI support and treatment for PWID.

## Methods

### Study design

This study used a pragmatic qualitative methodology to iteratively gather input from key knowledge users (expert consultation process) on the development of a comprehensive and evidence-based online stigma reduction education program. The study used an inductive qualitative approach informed by grounded theory methods. It aimed to answer the research question: What content and format should be used to develop an online stigma reduction education program for HCWs working with PWID? This study is guided by the Health Stigma and Discrimination Framework, which conceptualizes stigma as a multi-level process involving drivers, labeling, prejudicial attitudes, and discriminatory behaviors that ultimately influence health outcomes. The program was designed to improve HCWs’ knowledge of IDU, SSTI, injection practices, and health-related stigma, as well as to enhance their attitudes towards PWID through educational content on harm reduction, person-centered care, and nonjudgmental interactions [10].

Prior to expert consultations, a comprehensive review of the literature was conducted to understand the general characteristics of a stigma reduction education program to better support PWID in accessing SSTI services. The literature review aimed to identify the necessary topics to address and the timing and methods of delivery to HCWs. Preliminary program materials, including a detailed outline of the program topics organized into modules, were developed and presented to PWID and HCWs for input on content and format to inform the program development.

The qualitative design enabled participants to contribute insights and expertise on the content and format of the program. Semi-structured interviews with PWID allowed for open discussions on sensitive topics, informing the program’s content [19]. Focus groups with HCWs facilitated discussions and group brainstorming to shape the program’s development [20].

Ethical approval for this study was obtained from the Queen’s University Health Sciences & Affiliated Teaching Hospitals Research Ethics Board (HSREB), and the approval number is 6038401.

### Setting and participant recruitment

Participants were recruited from five communities (Hamilton, Toronto, Niagara, Kitchener-Waterloo, Ottawa) with populations ranging between 143,740 and 2,794,356 in Ontario, Canada [21]. The recruitment process started on 29^th^ April 2023 and ended on 7^th^ June 2023. The principal investigator (PI) partnered with several community-based healthcare organizations providing mental health and addiction services to distribute recruitment flyers to potential participants. The flyers were posted in common areas (e.g., waiting area, counselling room, examination room, and group/community room) and shared through newsletters and social media channels. The PI’s contact information was either provided to interested participants or consent was obtained for her to contact participants directly to confirm their eligibility to participate.

The PI conducted a telephone screening using a pre-defined script. During the phone screening, she explained the purpose of the study, confirmed the participants’ interest and eligibility, and obtained informed consent. The PI then scheduled a day and time for the individual interviews with PWID. A doodle poll was used to facilitate scheduling the HCWs’ focus groups. The eligibility criteria for PWID and HCWs are outlined in Table 1.

**Table 1:** Inclusion criteria for research participants.

PWID	HCW
• Adults aged 18–75 years old. • Self-reported history of injection drug use and homelessness within the last 5 years. • Accessing support services at a healthcare, social or community service organization. • Have access to a telephone to participate in a virtual interview. • Able to complete an interview in English Language. • Ability to provide voluntary informed consent *(assessed using the University of California, San Diego Brief Assessment of Capacity to Consent)* [22]. Participants must score an 8/10 to be included in the study.	• Be a healthcare worker (e.g., Physician, Nurse Practitioner, Registered Nurse, Social Worker, Community Health Worker, or Outreach Worker). • Have at least 2 years of experience in one of the following areas: harm reduction, mental health and addiction services, infectious disease, stigma education, health education. • Self-reported employment in a position that provides or oversees services for PWID. • Accessing support services at a healthcare, social or community service organization. • Have access to an internet-enabled device to participate in focus groups. • Able to complete the focus groups in English Language.

### Participants

**Fig 1:**
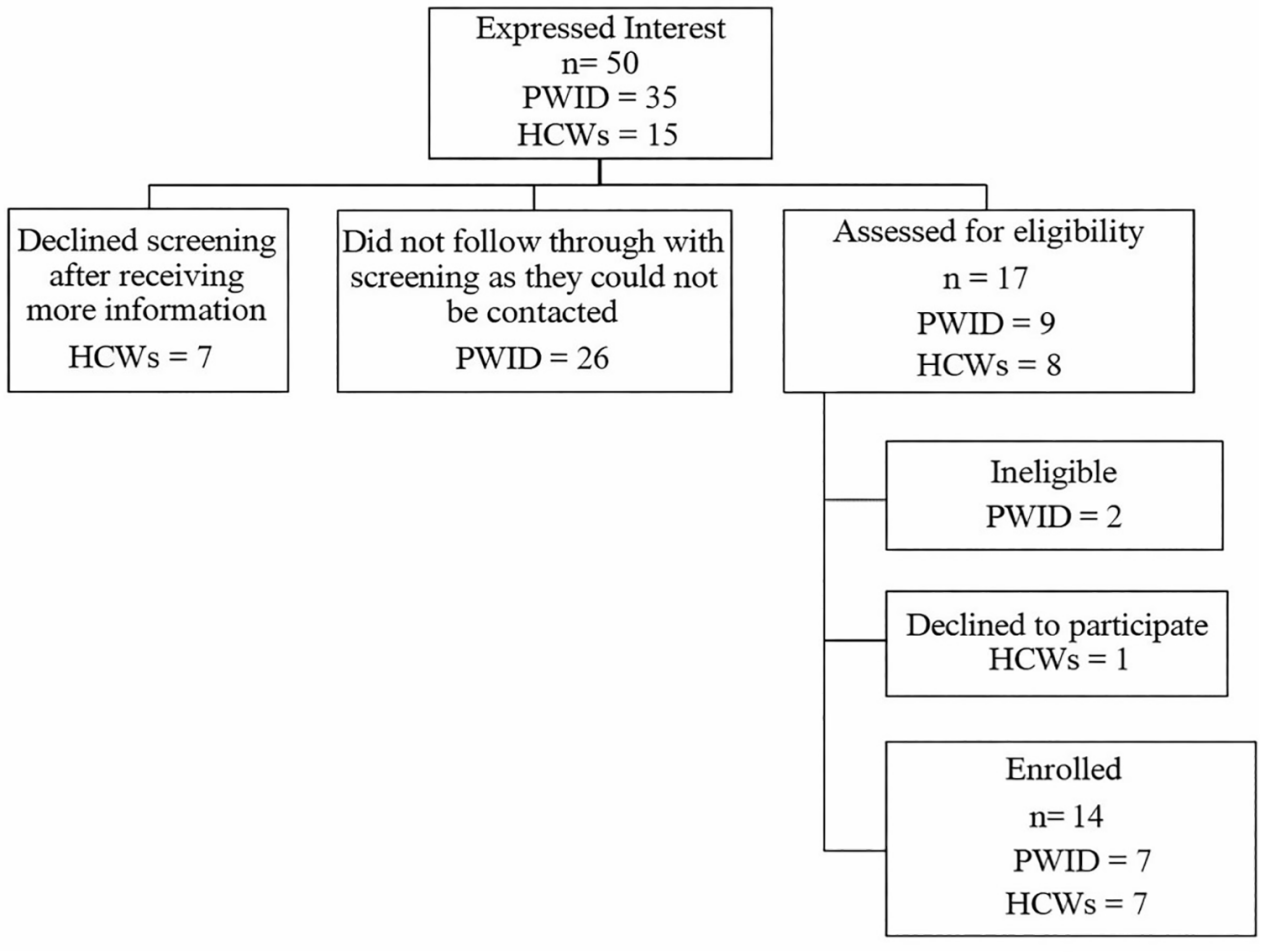
Flowchart of study recruitment and enrollment process.

A total of 50 individuals (35 PWID and 15 HCWs) expressed interest in the study. Twenty-six PWID did not complete the screening as they could not be contacted. Seven HCWs decided not to be screened after receiving more information about the study. Nine PWID and eight HCWs completed the telephone screening. After completing the screening process, three PWID were deemed ineligible due to an inability to provide voluntary informed consent. One HCW declined to participate due to a change in their schedule. Fourteen eligible participants (seven PWID and seven HCWs) returned a copy of the signed consent materials and were enrolled into the study. A flow chart summarizing the recruitment and enrollment process is presented in Fig 1.

### Data collection procedure

The data collection process was conducted iteratively. First, PWID individual semi-structured interviews were completed, followed by three focus groups with HCWs. Interviews and focus groups were conducted remotely using either telephone or Zoom [23], depending on participants’ preferences. The program development process was iterative, with four iterations of the expert consultation process. After each round of consultation, the prototype was revised and then reviewed by the experts.

Prior to the interview/focus group, participants were asked to reconfirm their consent, “Thank you for joining the interview today. Before we begin, I would like to reconfirm your agreement to participate in this study. Your participation in this study is voluntary, and you can stop your participation at any time by letting me know. Please reconfirm your agreement to participate in this study. Do you agree to participate in this study? Please answer Yes/No.”

#### PWID interviews.

The first step involved individual semi-structured interviews with seven PWID to seek input on content development. Interviews were conducted between April and May 2023. Before the interviews, participants completed a self-reported questionnaire. The questionnaire captured socio-demographic information (gender, age, history of injection drug use, history of skin and soft tissue infection, and housing situation). The PI facilitated all the interviews using a semi-structured guide with open-ended questions. Participants provided verbal consent before commencing the interview. During the interviews, a preliminary list of program topics, initially compiled based on the environmental scan [16,24,25], was shared with participants.

The interviewer took notes and carefully observed relevant non-verbal cues, which enabled her to adjust her approach to create a supportive and non-judgmental environment, responding with empathy and sensitivity. Prompts were used to clarify participants’ responses. Each interview ranged between 30–40 minutes. Interviews were digitally recorded and professionally transcribed.

#### HCWs focus groups.

One week before the first focus group, participants completed self-reported questionnaires capturing socio-demographic information (gender, age, employment, occupation, job title, job role, education level, and years of experience). No later than five days before each focus group session, participants were sent the preliminary program materials via email to review. Between June and August 2023, the PI facilitated three focus groups with the same seven HCWs in each focus group to further seek feedback on the program prototype via zoom using a semi-structured guide with open-ended questions to encourage participant discussions. Each focus group lasted approximately 30 min. All participants provided verbal consent before starting the discussion.

**Fig 2:**
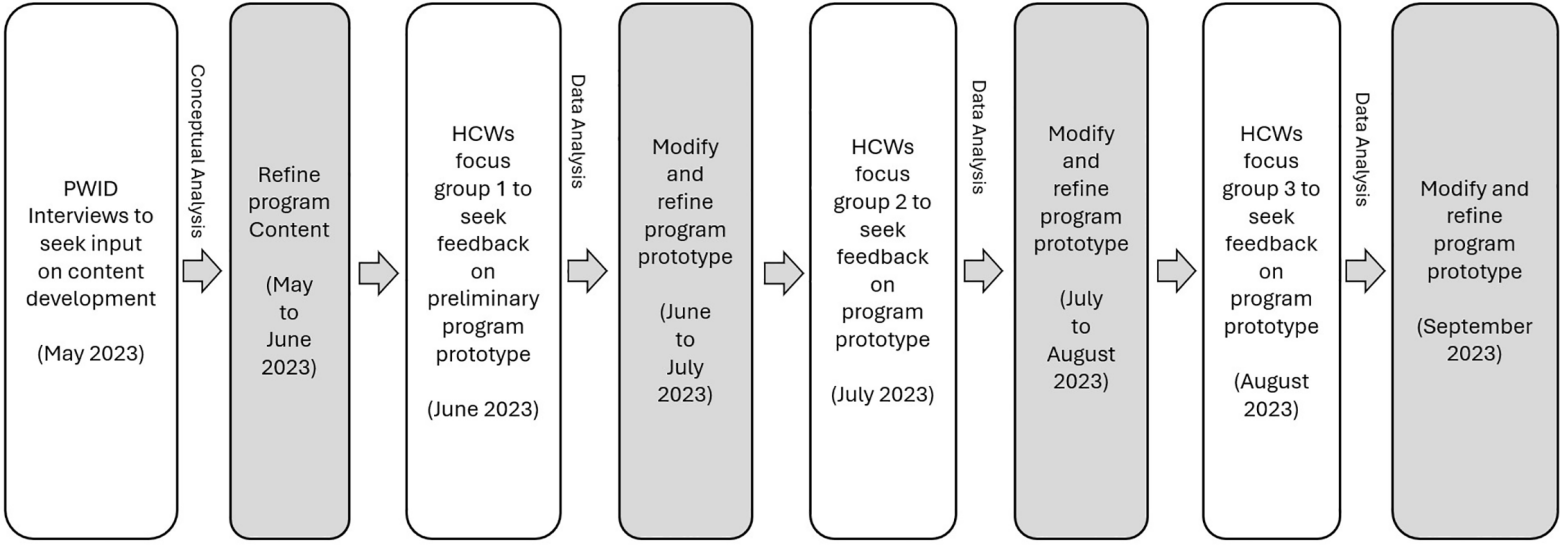
Flowchart of the program development process.

During the first focus group, the PI sought feedback on the content and activities to be included in the program and how they should be formatted and presented. Post-discussion feedback was collected anonymously through Zoom polls. Based on feedback obtained from participants, the program prototype was modified and refined. Subsequently, in the second focus group, feedback on detailed program content and format, including animated content videos and website user interface/user experience (UI/UX) design, was obtained. For the final focus group, a website was developed, and feedback on the website was sought. The website link “Project Holistique” and access were shared with the participants one week before the final focus group. The website was modified and refined based on feedback from participants. The final product was shared with all the authors for discussion and editorial revision. The flowchart of the program development process is provided in Fig 2.

### Data management and analysis

IBM SPSS Statistics 28 software was used to generate descriptive statistics for reporting sample characteristics, including mean, median, standard deviation, and range.

### PWID interview data analysis

Interview data were analyzed using conceptual content analysis [26] which allowed for an in-depth exploration of the data, specifically in identifying recurring patterns and emerging themes [27]. Conceptual content analysis focuses on determining the existence and frequency of concepts within the text. The PI reviewed the transcripts against the digital recordings for accuracy and anonymized participants by replacing their names with pseudonyms to protect their identities. Transcripts were then uploaded and organized into NVivo (Version 12, QSR International Pty Ltd, Melbourne, Australia) for efficient coding and data management.

- The analysis process began with the PI thoroughly reading all transcripts to comprehensively understand the content and context. Initial impressions, recurrent patterns, and key themes related to participants’ responses were noted.
- In the second stage, the PI identified concepts embedded in the data and developed an initial list of categories. These categories were cross-checked by the second and fourth authors to ensure alignment and accuracy.
- In the third stage, the PI further refined the initial categories by grouping similar concepts and creating subcategories under broader categories. This refinement process involved collaboration with the other authors to reach a consensus on the definitions and usage of categories and subcategories.
- In the fourth stage, the predefined categories were applied systematically to relevant sections of the transcripts. The PI conducted a line-by-line coding of the text, ensuring that each segment was coded according to the identified categories. The other authors further reviewed this coding process to ensure agreement and that discrepancies were resolved through discussion.
- A content map was developed to visualize the relationships between categories and subcategories. Different iterations of this content map were shared with the authors for discussion and refinement. Feedback from participants was incorporated to enhance the accuracy and relevance of the program content for the refined list of program topics.

### HCWs focus group data

After discussions on the program prototype, participants answered four questions via a Zoom poll and the responses were downloaded into an Excel spreadsheet to facilitate easy reference and analysis. The responses were categorized based on the questions asked. The PI manually tallied and calculated the number of “Yes” and “No” responses for each poll question, calculating the percentage of agreement or disagreement based on the total number of responses received. The specific qualitative responses to the question about program improvement were extracted and summarized, with an emphasis on recurring themes or suggestions. After analyzing the data, actionable recommendations for refining the program prototype were shared with all the authors for peer debriefing and validation. Based on the consolidated feedback obtained from each of the focus groups, the recommended improvements and modifications were integrated. The final product was the online stigma reduction education program.

### Trustworthiness

The strategies recommended by Lincoln and Guba [28] were used to ensure rigour. The PI kept a reflexive journal and field notes to document the research procedures [29]. The reflexive journal allowed the PI to examine any personal assumptions, clarify individual belief systems, and minimize potential biases [30]. The second and fourth authors assisted in eliminating potential biases by providing guidance throughout the process. Dependability and conformability were achieved by using an audit trail to connect the raw data and codes and track decisions made during coding and analysis. To ensure the accuracy of coding, data were repeatedly coded, reviewed, and re-coded [31]. To maintain the integrity of data analysis, the PI engaged in peer debriefing with all the authors for an external critique of evolving analytic conclusions and potential conceptualizations throughout the data analysis process [32].

## Results

### Characteristics of participants

#### PWID.

The sample of PWID (n = 7) included a diverse demographic profile. The participants, with a median age of 46 years, ranged in age between 28–62 years old. Gender distribution revealed five males, one female, and one non-binary participant. Regarding the duration of IDU, two participants reported 3–5 years of use, one reported 6–10 years, and four reported ≥11 years. All participants reported a history of wounds or SSTI. Housing situations varied, with four participants reporting being housed, one residing on the street or in encampments, and two residing in shelters.

#### HCWs.

Seven HCWs, with a median age of 42 years and a range between 37–54 years old, participated in the study. Four individuals reported working in community and primary health settings, while three reported roles in outreach and harm reduction. All seven participants identified themselves as frontline workers. Regarding educational background, two participants held postgraduate degrees, four participants held undergraduate degrees, and one held a certificate/diploma. Gender distribution included two males, four females, and one individual identifying as a demi girl. Occupations within the group varied, with three participants working as nurses, two in medical roles, one as a harm reduction community health worker, and one in an academic position. Specific roles at their places of employment encompassed one public health outreach nurse, one shelter health physician, two registered nurses, one health promoter focusing on Hepatitis C, one researcher and family doctor, as well as one harm reduction community health worker. Additionally, the participants reported varying years of experience in different professional areas, as presented in Table 2.

**Table 2:** Experience of healthcare workers in different areas.

	Mean (years)	Minimum (years)	Maximum (years)
**Harm Reduction**	9. 50	4	11
**Health Education**	9.17	5	11
**Infectious Disease**	7.33	1	11
**Mental Health and Addiction Services**	9.33	4	11
**Stigma Education**	8.83	4	11

### PWID interviews

Findings from interviews with PWID were organized into distinct categories aligned with the modules of the online stigma-reduction education program.

#### Module 1: Health-related stigma.

Participants consistently highlighted the significance and relevance of the proposed module 1 topics. PWID 106 expressed, “*I think they are all very important to include*,” while PWID 104 affirmed, “*Yes. Probably all of it is. I feel strongly*.” Key content areas identified were judgment and discrimination, and reluctance to access services.

- **Judgement and discrimination**
- Participants frequently encountered judgment and discrimination when seeking healthcare services due to their IDU history, underscoring the need for topics related to health-related stigma. PWID 101 stated, “*As soon as they know you are a drug user, you’re like lower than a piece of feces.*” PWID 103 added, “*They [HCWs] are scared of being nice*.” PWID 105 noted, “*They always judge you*… *But sometimes they discriminate against us, so people don’t want to go*”. PWID 107 stated, “*Like if you’re a drug user, they look down on you… They do not treat you well, they look down at you. Generally, they have negative stigma there too… “’I’m sure everybody thinks, who uses drugs is actually dangerous right here, right*”. These accounts reveal how stigmatizing attitudes lead to second-class treatment, making it essential to include these topics in the education program. By addressing judgment and discrimination, the program can help HCWs recognize and mitigate their biases, leading to better care for PWID.
- **Reluctance to access services**
- Participants highlighted their reluctance to access healthcare services due to stigma, further emphasizing the importance of addressing health-related stigma. PWID 101 remarked, “*It has made a big difference in my ability to access care*,” and PWID 107 echoed, “*Where should I go*?” PWID 105 articulated feelings of embarrassment, stating, “*It’s almost like, you know, coming to these health places, it was embarrassing…there’s a time in the beginning where it was almost like I was almost embarrassed to ask for stuff because of the way that people treat you here*.” These insights highlight the emotional toll and stigma attached to seeking healthcare services, which contribute to a reluctance to access available services.

#### Module 2: Skin and soft tissue infections.

Participants emphasized the relevance of including the proposed SSTI module topics. PWID 104 stated, “*Yes, they do. All of them are good to include*,” and PWID 105 added, “*Oh yeah, this is very important*.” Key areas identified were barriers to accessing services and self-treatment of SSTI. Barriers to accessing services was mentioned the most frequently by participants (n = 5).

- **Barriers to accessing services**
- Participants shared about the barriers they experience when attempting to access services for SSTI and expressed frustration over the lack of treatment received at health facilities due to their drug use history, further highlighting the need to include the topics for the education program. PWID 102 said, “*I went to the hospital, and they turned me away*” and PWID 103 mentioned, “*we’re not being treated again, we can’t go to the health clinic*”. PWID 103 conveyed a sense of hopelessness, suggesting that treatment might only be provided after their death, stating, “*You know, maybe they will treat it when I’m dead*”. PWID 105 described feeling judged when attempting to access healthcare facilities, making it difficult for them to seek treatment stating, “*It’s hard to go these health places cos of the judgement*”. PWID 106 noted the pervasive fear of judgment within healthcare settings, leading to avoidance of seeking treatment, stating, “*Yeah, people.. people fear to go for their treatment, I See that a lot. Even if you are going to hospital and you’re not using. They still think you are high, and they judge you. So we don’t go anymore*”.
- **Self-treatment of skin and soft tissue infections**
- Several participants described resorting to self-treatment for their SSTI due to barriers in accessing healthcare. PWID 105 stated, “*I try to clean it myself sometimes*,” indicating a reliance on self-care methods. PWID 107 shared a personal experience of contracting a Staph infection and opting to burst the wound themselves rather than seeking medical help because of the fear of discriminatory treatment due to stigmatization. These practices highlight the risks associated with self-treatment and the importance of including the topic in the education program.

#### Module 3: Harm reduction.

Participants supported the inclusion of harm reduction module topics. PWID 106 stressed the importance of incorporating harm reduction practices into healthcare to improve patient care and combat stigma stating, “*harm reduction in healthcare, it’s good for them to know the harm reduction practices and things like that, you know so that the care is better, and even the systems get better, treat clients specifically without judgement or stigma*”. PWID 107 highlighted the need for comprehensive training in stigma reduction for all healthcare professionals, including doctors, nurses, social workers, and personal support workers (PSW) stating “*Oh my God, yes, any doctor should get training in that, yes. OK, so I mean, not just doctors, right? like nurses, whoever social workers too, all of them healthcare workers yeah, even PSW, I used to work as a PSW so it’s like I know this. Everyone should get training*”.

#### Module 4: Stigma reduction.

Participants supported the inclusion of stigma reduction topics. Three subject areas were identified: ‘HCWs education’, ‘judgment and discrimination’, and ‘reluctance to access services.’

- **HCWs education**
- Participants emphasized the positive impact of stigma reduction training for HCWs on their ability to provide care and support for SSTI, highlighting the importance of including the module topics in the education program. PWID 105 shared a positive experience with a nurse who did not exhibit stigmatizing attitude during their visits to a healthcare facility stating, “*There’s this place that I have been going and the Nurse there, I’ve never feel any kind of stigma that I’m coming here*”. They also acknowledged the value of stigma reduction training for HCWs, suggesting that such training contributes to improved care “*And I think that’s better there because they have the training, like what you are trying to do*”. The participant also expressed optimism that stigma reduction training for healthcare workers could lead to a decrease in stigma “*So, if other people get the training and do the job and they do it very well. So, there will be less stigma*”.
- **Judgement and discrimination**
- Participants shared that they frequently encounter judgment and discrimination due to stigma, highlighting the necessity to include the topics on stigma-reduction. PWID 104 expressed frustration over being labelled as a “junkie” by HCWs, emphasizing the harmful effects of such language, stating, “*Not like I take issue with like changing my language but like they say that I’m a junkie. Like that’s the words they use and like that gets me mad. If you’re like saying thats, it’s like stigma and discriminatory*”. This sentiment highlights the participant’s distress at being labelled in a stigmatizing manner, recognizing the detrimental impact of such language on their well-being.
- The participant expressed feeling powerless in correcting HCWs who use stigmatizing language, stating, “*Like I don’t like workers calling me that and we can’t correct them*.” This statement highlights the pervasive nature of stigma within healthcare settings and the challenges encountered by individuals in advocating for respectful treatment. It speaks to a broader issue of power dynamics and discrimination, wherein PWID may feel marginalized and unable to assert their rights to dignified care.
- **Reluctance to access services**
- Participants highlighted that health-related stigma significantly influenced their decision-making regarding healthcare access. PWID 103 stated, “*That’s why we’re not going in all the time to get services*”, and PWID 107 stated, “*That’s why a lot of people don’t go to the hospital or clinics. That’s why a lot of people don’t get looked at. Looked after because they fear stigma*.” This sentiment underscores the impact of stigma on individuals’ willingness to seek medical attention for their SSTI. Furthermore, PWID 107 likened the experience of seeking healthcare to a circus, expressing discomfort and scrutiny. They stated, “*They don’t want to be like that, like a circus, you know.*” This further emphasizes the negative perceptions and experiences associated with accessing services in the presence of stigma for PWID highlighting the importance of including the topics to the education program.

#### HCWs focus groups.

Participants reached a consensus regarding the content and format of the stigma reduction education program. Following the discussions, responses were positive, with participants providing feedback on the development of the program prototype. Participants suggested keeping the modules concise to maintain engagement, recommending a mix of text, educational videos, and interactive elements to cater to different learning styles. They emphasized that the website layout should be user-friendly, with clear navigation and accessible resources to enhance the user experience. Additionally, participants appreciated the inclusion of check-in questions to encourage reflection and reinforce learning, and they valued the website’s role as a hub for resources. They also highlighted the importance of making the education program self-paced and easily accessible at all times, allowing HCWs to complete the training at their own pace and revisit materials as needed. This approach ensures that the program is flexible and accommodates the varying schedules and commitments of HCWs, ultimately contributing to its effectiveness in reducing stigma and improving SSTI care for PWID.

When asked for suggestions to further improve the program, participants provided valuable insights. For example, HCW 102 recommended, “*Include real experiences of PWID using quotes from PWID interviews*”. HCW 103 proposed “*emphasize that harm reduction is not a medical intervention to treating drug use, and PWID may not be in a position to remain abstinent. Emphasizing PWID autonomy and listening*”, HCW 105 suggested, “*acknowledge PWID lived experiences and direct PWID to other wrap-around supports in the community emphasizing drug-user-led organizations*”. One participant gave positive feedback about the education program stating, “*All is good, great initiative*”.

Concerning the feedback to further improve the program, HCW 101 from focus group 2 suggested: “*including a reflection question: what are your own values and beliefs about PWID*”. HCW 102 advised “*remove the word Addiction from “Dependence or Addiction” slide*”. HCW 103 suggested “*make the animation of trainer and participants gender diverse*.” HCW 105 recommended “*use words like “sterile or single-use equipment” instead of “clean equipment,” Change Naloxone Kit images to reflect Canadian standards*.”

Similarly, all participants of focus group 3 unanimously agreed on the final product which was the education program on a website. They expressed positive sentiments, with HCW 101 commending the work stating, “*great work*”, “*nothing else, good luck with the rest of the study*”. HCW 102 and HCW 103 both indicated satisfaction, with HCW 102 stating “*all good*” and HCW 103 adding that everything looked good and complete “*looks good, nothing else to include*”. HCW 106 commended the website, stating, “*great website*”, while HCW 107 emphasized the significance of the study, stating, “*looks great good luck, definitely a much-needed study*”.

The iterative refinement process for the program content across consultations with PWID and healthcare workers is summarized in Table 3.

**Table 3:** Iteration process for the development of the program content.

Module	Preliminary Program Content	Feedback from PWID Consultations (content analysis)	Include (Yes/No)	Feedback from HCWs focus group 1	Include (Yes/No)	Feedback from HCWs focus group 2	Include (Yes/No)	Feedback from HCWs focus group 3
1	Overview of stigma in healthcare settings Overview of stigma as a barrier for PWID communities accessing services Outline some common experiences and barriers PWID encounters when accessing health services Outline the relationship between health-related stigma and poor health outcomes Outline some common myths on PWID and IDU Outline the person-first approach as opposed to pathology- first approach	Include all topics Include information on stigma due to race and sexual orientation Include information on stereotypes	Yes No. Rationale: The purpose of the stigma-reduction education program is to address the health-related stigma due to injection drug use that PWID experience in health settings so as to improve access to skin and soft tissue infections support and treatment Yes	Include all topics	Yes	Content is appropriate for achieving the program’s purpose Gender diversity of the trainer and participant animation Include introductory question: What are your own values and beliefs about PWID	Yes Yes Yes	Yes
2	Overview of why PWID often develops wounds and skin and soft tissue infections Overview of the connection of stigma and complications of skin and soft tissue infections Outline why PWID may self-treat/ pro-long seeking out care and how to empathize with the patient when providing treatment or support instead of blaming Outline some common drug-specific language Outline some common drug use habits Overview of the intersection of homelessness and injection drug use	Include all topics Move intersection of homelessness and injection drug use to Module 1	Yes Yes	Include all topics Include real experiences of PWID using quotes from PWID interviews	Yes Yes	Content is appropriate for achieving the program’s purpose Use words like “sterile or single use equipment” instead of “clean equipment”	Yes Yes	Yes
3	Overview of harm reduction principles Overview of harm reduction in healthcare – specific to injection drug use Outline harm reduction methods – specific to SSTI	Include all topics Include information on safer supply programs and drug testing Include information on frequent infection testing for PWID	Yes Yes Yes	Include all topics Emphasize that harm reduction is not a medical intervention to treating drug use, and PWID may not be in a position to remain abstinent	Yes Yes	Content is appropriate for achieving the program’s purpose Change Naloxone Kit images to reflect Canadian standards Remove the word Addiction from “Dependence or Addiction” slide	Yes Yes Yes	Yes
4	Outline how healthcare workers make a difference Outline stigma reduction practices and behaviours Overview of non-judgmental interaction Overview of person-centered approach techniques Overview of the importance of language use and stigma and how to incorporate respectful language Outline practical, behavioral ways of not perpetuating stigma	Include all topics Include information on client confidentiality and privacy	Yes Yes	Include all topics. Emphasizing PWID autonomy and listening Acknowledgment of PWID lived experiences and directing PWID to other wrap-around supports in the community with an emphasis on drug-user-led organization	Yes Yes Yes	Content is appropriate for achieving the program’s purpose	Yes	Yes

### Comparison of interviews with PWID and HCWs focus groups

The results from interviews with PWID and focus groups with HCWs revealed both commonalities and differences in their perceptions of stigma as a barrier to SSTI care and the impact of the stigma reduction program. Both groups recognized the presence of stigma in healthcare, with PWID sharing personal experiences of judgment and discrimination, while HCWs acknowledged the issue and emphasized the need for stigma reduction education. Both groups provided feedback on the content. PWID shared personal experiences of stigma and discrimination in healthcare settings and HCWs suggested incorporating real-life examples of PWID to enhance relatability. Both also emphasized the importance of including content on harm reduction, with PWID highlighting the role of harm reduction in reducing stigma and HCWs viewing it as essential for supporting PWID without imposing abstinence. Differences emerged in how each group perceived HCWs’ attitudes and barriers to accessing care. PWID reported feeling dehumanized and judged, often resorting to self-treatment due to fear of mistreatment when accessing SSTI care, while HCWs saw themselves as empathetic and acknowledged the need for systemic change. Incorporating feedback from both groups is essential for developing an effective stigma reduction program.

### Overview of the developed educational program

The course included four modules: (1) Stigma and discrimination in healthcare settings, (2) Skin and soft tissue infections and injection drug use, (3) Harm reduction, and (4) Stigma reduction practices. Each module required approximately 1–2 hours to complete and included a combination of educational videos, textual content, practical scenarios, and assessment questions.

The first module introduces the concept of stigma and its underlying drivers, discusses the presence of stigma in healthcare settings, and highlights common experiences and barriers faced by PWID when accessing healthcare services. It also examines the relationship between health-related stigma and poor health outcomes and introduces person-first approaches to care.

The second module focuses on common skin and soft tissue infections (SSTIs) experienced by PWID. It discusses the relationship between stigma and delayed treatment of SSTIs, introduces commonly used drug-related terminology, and reviews common injection practices.

The third module provides an overview of harm reduction principles and practices related to injection drug use and SSTI prevention.

The final module focuses on practical stigma reduction strategies, including non-judgmental communication, person-centred care approaches, and the importance of respectful language in reducing stigma in healthcare settings.

## Discussion

This study set out to determine the content and format for the development of an online stigma reduction education program for HCWs working with PWID. While the importance of educational interventions for stigma reduction is well-documented in the literature [33–37], our study stands out due to its tailored focus on addressing the stigma encountered by PWID when accessing services for SSTI. This targeted approach has not been extensively explored previously. It is imperative that professionals who support PWID have access to information and education to provide compassionate and non-judgemental care. By tailoring our intervention to this specific context, we can more effectively address the unique challenges and barriers experienced by PWID when seeking healthcare services. Thus, this study provides initial insights that may contribute to the development of educational approaches addressing stigma in this context. The expert consultations conducted in our study, including individual interviews with PWID and focus groups with HCWs, provided invaluable insights into the development of a tailored, online stigma-reduction education program. Through iterative feedback, participants contributed to shaping the content and format of the program, ensuring its relevance in addressing stigma in healthcare settings. A significant finding from our study was the clear identification of both the essential content and effective format for an online stigma reduction education program for HCWs working with PWID.

The study revealed that including modules on health-related stigma, SSTI, harm reduction, and stigma reduction is crucial. These topics were highlighted as vital by both PWID and HCWs to address stigma and improve healthcare access and outcomes for PWID. Additionally, the study underscored the importance of a user-friendly and easily accessible format for the education program. Focus groups participants suggested that the modules be concise and engaging, using a mix of text, educational videos, and interactive elements. The layout should have clear navigation and accessible resources, check-in questions to encourage reflection. The program’s accessibility at all times allows HCWs to complete the training at their own pace. These insights informed the development and refinement of the program prototype, including recommendations regarding content areas and delivery format for an online stigma reduction education program.

The exploration of stigma as a barrier to accessing SSTI healthcare services among PWID has received limited research attention [38]. Most of the existing literature has reported stigma as a restriction to accessing primary care [39], for HIV prevention [40], and hepatitis C virus (HCV) prevention [41] among PWID. Participants in the current study, particularly those with lived experience of IDU, shared personal accounts of encountering judgment and discrimination, leading to their reluctance to seek available SSTI services due to fear of stigma. These findings emphasize the importance of our study in addressing the gap in online stigma reduction training for HCWs working with PWID. By equipping providers with the knowledge and skills to approach care with empathy, understanding, and without judgment, we can create a more supportive and inclusive healthcare environment for PWID, including better care and treatment for SSTI.

Online stigma reduction education programs offer cost-effective solutions compared to traditional face-to-face interventions [42]. Once established, these programs have high levels of sustainability and scalability, as they can be readily expanded to reach a wider audience without incurring significant additional expenses [14]. Therefore, this study lays the groundwork for the next phase of research, which involves conducting a feasibility study to pilot test the developed online stigma reduction education program.

### Strengths and limitations

The study provides valuable insights to develop an online stigma reduction education program for HCWs working with PWID and lays the groundwork for future research endeavours aimed at developing effective interventions to promote stigma-free care for PWID accessing SSTI services. However, there are important limitations to consider. A limitation of this study is that the measures used primarily captured the attitudinal dimension of stigma and therefore may not fully reflect the behavioral or structural manifestations of stigma experienced by PWID in healthcare settings. Additionally, the recruitment strategy solely focused on one Canadian province (Ontario) which may limit the applicability of the education program to other regions with different healthcare systems, cultural contexts, and types of stigma experiences [16,43–45]. A more diverse sample, including participants from multiple provinces or countries, would have enhanced the applicability of findings. The sample size of this study was small (n = 14), which, with the voluntary participation, may have introduced selection bias. Furthermore, considering the qualitative nature of the study, the findings based on self-reported data may be influenced by social desirability bias, recall bias or willingness of participants to share their experiences or feedback openly.

## Conclusion

This study has informed the development of an online stigma reduction education program for HCWs working with PWID. The education program is aimed to equip HCWs with the knowledge and skills necessary to reduce the stigma associated with IDU, thereby improving access to SSTI support and treatment for PWID. Insights obtained from key knowledge users, including PWID and HCWs facilitated the design of content and format, and recommendations informed by lived experiences and expert suggestions. The resulting program outlines key educational modules intended to support HCWs in developing knowledge and skills related to stigma awareness, harm reduction, and compassionate care for PWID. Future research will require to evaluate the feasibility, effectiveness, and potential impact of the program.

## Supporting information

S1 File HCW focus group guide.docx. Focus group guide.
(DOCX)
