# Supplementary material for: An online stigma reduction education program for healthcare workers working with people who inject drugs: Development
Source: PLoS One. 2026 Apr 24;21(4):e0347309. doi: 10.1371/journal.pone.0347309 (PMC13108893; doi:10.1371/journal.pone.0347309)
Supplement: S1 File — Focus group guide. (DOCX) [file pone.0347309.s001.docx]

Focus Group Guide

This study seeks to obtain your input on the development of an online stigma reduction education program prototype for healthcare workers working with people who inject drugs.

This focus group:

- will be approximately 40 minutes in length
- will seek feedback from you on the program’s content and structure.
- will obtain feedback using the Zoom polling feature
- is an iterative process, and this is the (insert #) focus group session

| Thank participants for joining today; reconfirm verbal consent; review the guiding principles of confidentiality; encourage participants to share freely whatever they wish. | |
| --- | --- |
| I hope you have had a chance to review the program materials that were sent ahead of our discussion today. Confirm that participants have reviewed the materials. | |
| **Feedback on prototype development (focus group 1, 2, 3)** | |
| 1. In your opinion, how appropriate is the content of the education program in achieving the program’s purpose? *Allow for 8 minutes of discussion* | **Zoom poll question**: Is the suggested content appropriate for achieving the program’s purpose?  Yes No  Some changes required |
| 2. What are your thoughts regarding the format/layout of the program?  *Allow for 8 minutes of discussion* | **Zoom poll question**: Is the suggested format/layout of the program appropriate?  Yes No  Some changes required |
| 3. What are your thoughts on the length of the program?  *Allow for 8 minutes of discussion* | **Zoom poll question**: Is the suggested length of the program appropriate?  Yes No |
| 4. What are your thoughts regarding areas of improvement for the program?  *Allow for 8 minutes of discussion* | **Zoom poll question**: Is there anything else that could be done to improve the program?  Yes No  Some changes required |
| **Focus Group Exit** | |
| Thank you so much for providing feedback on the development of the education program prototype. Your feedback will help me to develop an evidence-informed and comprehensive education program for healthcare workers working with people who inject drugs. I have learned a lot from you, and I appreciate the time you took out today.  -End Focus Group- | |
